# A new name and seventeen new combinations in the *Magnolia* (Magnoliaceae) of China and Vietnam

**DOI:** 10.1186/1999-3110-54-53

**Published:** 2013-11-04

**Authors:** Chris Callaghan, Siak-Khoon (SK) Png

**Affiliations:** Australian Bicentennial Arboretum, P.O. Box 88, Penshurst, NSW 2222 Australia

**Keywords:** *Magnolia fansipanensis*, Magnolioideae, *Manglietia*, *Michelia*, new combinations, new name, *Yulania*

## Abstract

**Background:**

A new name is proposed and seventeen new combinations are made as a result of the previous reduction of the remaining genera of subfamily Magnolioideae (Magnoliaceae) into the genus *Magnolia*.

**Results:**

The replacement name *Magnolia fansipanensis* is proposed for *Manglietia crassifolia* Q. N. Vu et al., since its transfer to *Magnolia* would create an illegitimate later homonym of the fossil name *M. crassifolia* Göpp. A further 17 new combinations are made to transfer the following taxa to *Magnolia*: *Manglietia guangzhouensis* A. Q. Dong et al., *M. kaifui* Q. W. Zeng & X. M. Hu, *M. lawii* N. H. Xia & W. F. Liao, plus *Michelia concinna* H. Jiang & E. D. Liu, *M. jianfenglingensis* G. A. Fu & K. Pan, *M. viridipetala* Y. W. Law et al., *M. wuzhishangensis* G. A. Fu & K. Pan, *M. xianianhei* Q. N. Vu and *Yulania carnosa* D. L. Fu & D. L. Zhang, *Y. cuneatofolia* T. B. Chao (probably Zhao) et al., *Y. dabieshanensis* T. B. Zhao et al., *Y. dimorpha* T. B. Zhao & Z. X. Chen, *Y. fragarigynandria* T. B. Zhao et al., *Y. shirenshanensis* D. L. Fu & T. B. Zhao, *Y. shizhenii* D. L. Fu & F. W. Li, *Y. verrucata* D. L. Fu et al. and *Y. xinyangensis* T. B. Zhao et al.

**Conclusions:**

The transfer of the above taxa to *Magnolia* is necessary following the present almost universal recognition of Magnolioideae as one of two monogeneric subfamilies within Magnoliaceae.

**Electronic supplementary material:**

The online version of this article (doi:10.1186/1999-3110-54-53) contains supplementary material, which is available to authorized users.

## Background

The authors of a number of new magnoliaceous taxa from China and Vietnam described in recent years have followed the treatment of Magnoliaceae in the Flora of China (Xia et al. 2008), where *Manglietia* and *Michelia* are retained and *Yulania* is reinstated as segregate genera in subfamily Magnolioideae. In the present account, 17 new combinations are made and one new name is proposed in accordance with the relegation of all remaining genera of subfamily Magnolioideae into *Magnolia* by Figlar (2000), Figlar and Nooteboom (2004).

## Methods

The new combinations and new name proposed in this paper are made in compliance with the rules and recommendations of the 2011 International Code of Nomenclature for algae, fungi and plants (ICN), known as The Melbourne Code (McNeill, Turland et al. 2012), in particular ICN Article 41 and Recommendation 41A in respect of new combinations and replacement names.

## Results and discussion

The problematic past 150 year history of the family Magnoliaceae is too lengthy to detail here. Suffice it to say that with the sinking of all remaining genera of the previously contentious subfamily Magnolioideae into *Magnolia*, that there is at last relative stability in this subfamily that would appear will require minimal refinement or disruptive changes in the future. For the most comprehensive accounts of past changes ultimately leading to almost universal acceptance of the family Magnoliaceae as consisting of two subfamilies, Liriodendroideae represented only by *Liriodendron*, and the now monogeneric Magnolioideae comprising all its former genera, including *Michelia* and *Manglietia*, now under *Magnolia*, the reader is referred to the historical records provided by Richard (Dick) Figlar, the former president of Magnolia Society International, that have appeared in various publications since the first International Symposium on Family Magnoliaceae (Figlar 2000 and particularly Figlar 2006 Figlar 2009 Figlar 2012).

As Figlar makes clear in his later papers, the advent of molecular DNA sequencing data, in conjunction with on-going morphological study, especially of living plants, has allowed critical analysis of the taxonomic relationships of subfamily Magnolioideae taxa during the last ca. 20 years, that has surpassed any understanding of the classification of the subfamily that had accrued in the preceding more than a century of mainly morphological research.

The findings of the studies of numerous researchers that are most pertinent to the present paper (DNA analysis by Azuma et al. 1999,2000,2001 Kim et al. 2001 Nie et al. 2008 Wang et al. 2006; morphological studies by Figlar plus others; morphological studies by Figlar 2000 Figlar and Nooteboom 2004), have provided convincing evidence that both *Manglietia* and *Michelia* are nested within the genus *Magnolia*, the former under Subgenus *Magnolia* Section *Manglietia* and the latter under Subgenus *Yulania* Section *Michelia*, Subsection *Michelia*.

In accordance with the above findings, a number of new combinations are made here, representing three species of *Manglietia*, five species of *Michelia* and nine species of *Yulania* named and described between 2008 and 2012 that are now transferred to *Magnolia*. Also, since its epithet in *Magnolia* is unavailable, *Manglietia crassifolia*, named in 2011, is renamed as *Magnolia fansipanensis* to reflect its occurrence on the north-eastern slopes of Mount Fansipan, the ‘roof of Indo-China’.

**Magnolia carnosa** (*D. L. Fu & D. L. Zhang*) *C. B. Callaghan & SK Png* **comb. nov.**

**Basionym:** *Yulania carnosa* D. L. Fu & D. L. Zhang. *In*: D.L. Fu, D. L. Zhang, L. F. Wen, et al., Bull. Bot. Res. 30(4): 385, fig. 1 (2010).

TYPE: China, Sichuan, Beichuan County, alt. 1200 m., secondary forests, 14 Mar. 2001, *D. L. Fu 200103141* (holotype: **CAF**).

**Magnolia concinna** (*H. Jiang & E .D. Liu*) *C. B. Callaghan & SK Png* **comb. nov.**

**Basionym:** *Michelia concinna* H. Jiang & E. D. Liu. *In*: Annales Botanici Fennici 45(4): 290, fig. 1 (2008).

TYPE: China, Yunnan, Kunming Arboretum, alt. 1990 m, 22 March 2006, *H. Jiang 03942* (holotype: **KUN**; isotypes: **H**, **PE**, **YAF**). [transplanted from an unknown habitat in SE Yunnan].

Note – *Magnolia concinna* Y. W. Law & R. Z. Zhou. In: Liu et al. (2004) 44, is an invalid name for a different taxon, as no Latin description or diagnosis was given (ICN Art. 39.1) and no type was indicated. (ICN Art. 40.1).

**Magnolia cuneatifolia** (*T. B. Chao, Z. X. Chen & D. L. Fu*) *C. B. Callaghan & SK Png* **comb. nov.**

**Basionym:** *Yulania cuneatofolia* T. B. Chao, Z. X. Chen & D. L. Fu. *In*: D. L. Fu et al. Bull. Bot. Res. 30(6): 643, fig. 2 (2010).

TYPE: China, Henan, Zhengzhou, 24 Mar. 2005, *T. B. Zhao et al. 200503241* (holotype: **HEAC**; isotype: **CAF**); ibid. 18 Sept. 2005, *T. B. Zhao & Z. X. Chen* (**CAF**).

Note 1 – There is an apparent orthographical error in the original epithet which should be ‘*cuneatifolia*’, as this Latin compound should be formed by the connecting vowel -*i-*. (ICN Art. 60.1 – “the original spelling of a name or epithet is to be retained, except for the correction of typographical or orthographical errors”). Note 2 – There is a probable error in the lead author’s name which may be T. B. Zhao as for the specimen collector’s name. Note 3 – While the paper in which *Yulania cuneatofolia* is named as a new species is titled “Two new species of *Yulania* Spach from Hubei Province”, this species is only noted in that paper as occurring in Henan Province, as for the type specimens above.

**Magnolia dabieshanensis** (*T. B. Zhao, Z. X. Chen & H. T. Dai*) *C. B. Callaghan & SK Png* **comb. nov.**

**Basionym:** *Yulania dabieshanensis* T. B. Zhao, Z. X. Chen & H. T. Dai. *In*: H. T. Dai, D. W. Zhao, J. Li, et al., J. Xinyang Normal Uni. (Nat. Sci. Ed.) 25(3): 334, fig. 2 (2012).

TYPE: China, Henan, Jigongshan, 24 Feb. 1999, *Tian-bang Zhao 992241* (holotype: **HEAC**, fl.); ibid. 18 July 1999, *997181* (**HEAC**, fol.).

Note – The type collection was made on Mount Jigongshan in the Dabieshan mountain range straddling the border of Henan and Hubei Provinces, hence the specific epithet.

**Magnolia dimorpha** (*T. B. Zhao & Z. X. Chen*) *C. B. Callaghan & SK Png* **comb. nov.**

**Basionym:** *Yulania dimorpha* T. B. Zhao & Z. X. Chen. *In*: H. T. Dai, J. Li, D. L. Fu, et al., J. Xinyang Normal Uni. (Nat. Sci. Ed.) 25(4): 483, fig. 1 (2012).

TYPE: China, Henan, Xinyang Xian, Mt. Dabieshan, 25 April, 1994, *T. B. Zhao et al*. 944251 (holotype: **HEAC**).

**Magnolia fansipanensis** *C. B. Callaghan & SK Png* **nom. nov.**

**Replaced synonym:** *Manglietia crassifolia* Q. N. Vu, N. H. Xia & Y. K. Sima. *In*: Novon 21(3): 375, fig. 1 (2011), non *Magnolia crassifolia* Göppert (1852) 277 [fossil taxon].

TYPE: Vietnam, Lao Cai Province, Sa Pa District, Hoang Lien National Park, alt. 1890 m, 18 Dec. 2009, *V. Q. Nam 181209.3* (holotype: **VNF**; isotype: **IBSC**).

Note 1 – The replacement name *Magnolia fansipanensis* is proposed for *Manglietia crassifolia* because its transfer to *Magnolia* would create a later homonym to the fossil taxon *Magnolia crassifolia* Göpp. (ICN Art. 53.1, also ICN Art. 11.8 *Note 5* – “in accordance with Art. 53, later homonyms are illegitimate whether the type is fossil or non-fossil”). Note 2 – The species is renamed after Vietnam’s highest mountain, Mount Fansipan, which the present authors climbed in 2007 (Callaghan 2008), and 2010, and on whose north-eastern slopes the species occurs in small numbers between 1800–2000 m. Note 3 – As the lead author and the collector are the same person (Vu Quang Nam), there would appear to be an irregular citation in the name of this collector of the nomenclatural type and its duplicate. While the use of a person’s given name is customary as a primary form of address in Vietnam, its use in an international scientific journal is potentially confusing.

**Magnolia fragarigynandria** (*T. B*. *Zhao*, *Z*. *X*. *Chen & H*. *T*. *Dai*) *C*. *B*. *Callaghan & SK Png* **comb. nov.**

**Basionym:** *Yulania fragarigynandria* T. B. Zhao, Z. X. Chen & H. T. Dai. *In*: H. T. Dai, D. W. Zhao, J. Li et al., J. Xinyang Normal Uni. (Nat. Sci. Ed.) 25(3): 334, fig. 1 (2012).

TYPE: China, Henan, Changyuan County, *Tian*-*ban Zhao et al*. *200303115* (holotype: **HEAC**, fl.).

**Magnolia guangzhouensis** (*A*. *Q*. *Dong*, *Q*. *W*. *Zeng & F*. *W*. *Xing*) *C*. *B*. *Callaghan & SK Png* **comb. nov.**

**Basionym:** *Manglietia guangzhouensis* A. Q. Dong, Q. W. Zeng & F. W. Xing. *In*: A. Q. Dong et al., Nordic J. Bot. 27: 339 (2009).

TYPE: China, Guangdong, Guangzhou, Conghua, Mount Dalingshan, in evergreen broad-leaved forests, ca. 750 m. 14 June, 2007, *An*-*qiang Dong 1184* (holotype: **IBSC**).

**Magnolia jianfenglingensis** (*G*. *A*. *Fu & K*. *Pan*) *C*. *B*. *Callaghan & SK Png* **comb. nov.**

**Basionym:** *Michelia jianfenglingensis* G. A. Fu & K. Pan. *In*: Bull. Bot. Res., Harbin 32(3): 257, fig. 1 (2012).

TYPE: China, Hainan Island, Ledong County, Jianfengling, ca. 800 m. 22 August, 1995, *Guo*-*ai Fu 11338* (holotype: **HFB**, fl.); ibid. *Guo*-*ai Fu 8144* (**HFB**, fr.).

**Magnolia kaifui** (*Q*. *W*. *Zeng & X*. *M*. *Hu*) *C*. *B*. *Callaghan & SK Png* **comb. nov.**

**Basionym:** *Manglietia kaifui* Q. W. Zeng & X. M. Hu. *In*: X. M. Hu et al., Pakistan J. Bot. 43(5): 2270, figs. 1, 2 (2011).

TYPE: China, Yunnan, Luchun County, Mount Huanglianshan, monsoon evergreen forests, alt.1300–2000 m, 12 May 2004, *Qing*-*Wen Zeng 88* (holotype and isotype: **IBSC**).

**Magnolia lawii** (*N*. *H*. *Xia & W*. *F*. *Liao*) *C*. *B*. *Callaghan & SK Png* **comb. nov.**

**Basionym:** *Manglietia lawii* N. H. Xia & W. F. Liao. *In*: Liao and Xia, Nordic J. Bot. 27(1): 1, ill. (2009).

TYPE: China, Yunnan, Maguan County, mixed woods of Fagaceae and Lauraceae, alt.1500–2000 m. 16 May 1982, *Y*. *H*. *Liu 7032* (holotype: **IBSC**).

**Magnolia shirenshanensis** (*D*. *L*. *Fu & T*. *B*. *Zhao*) *C*. *B*. *Callaghan & SK Png* **comb. nov.**

**Basionym:** *Yulania shirenshanensis* D. L. Fu & T. B. Zhao. *In*: D. X. Zhao, T. B. Zhao & D. L. Fu,: D. X. Zhao, T. B. Zhao & D. L. Fu, : D. X. Zhao, T. B. Zhao & D. L. Fu,^2011^ Int’l Conf. Agricult. Nat. Res. Eng.: Adv. Biomed. Eng. 3–5: 91, fig. 1 (2011).

TYPE: China, Henan, Lushan County, Shirenshan, 26 Mar. 2000, *T*. *B*. *Zhao et al*. *200003261* (holotype: **HNAC**, fl.); ibid. 25 Aug. 2000, *D*. *L*. *Fu & T*. *B*. *Zhao 200008251* (**HNAC**, fol., br.).

**Magnolia shizhenii** (*D*. *L*. *Fu & F*. *W*. *Li*) *C*. *B*. *Callaghan & SK Png* **comb. nov.**

**Basionym:** *Yulania shizhenii* D. L. Fu & F. W. Li. *In*: D. L. Fu, D. L. Zhang, L. F. Wen, et al., Bull. Bot. Res. 30(4): 387, fig. 2 (2010).

TYPE: China, Sichuan, Chengdu City, 16 Mar. 2001, *D*. *L*. *Fu 200103161* (holotype: **CAF**, fl., juv. fol.); ibid. 11 Sept. 2000, *D*. *L*. *Fu 200009112* (**CAF**, br., fol.).

**Magnolia verrucata** (*D*. *L*. *Fu*, *T*. *B*. *Zhao & S*. *S*. *Chen*) *C*. *B*. *Callaghan & SK Png* **comb. nov.**

**Basionym:** *Yulania verrucata* D. L. Fu, T. B. Zhao & S. S. Chen. *In*: Bull. Bot. Res. 30(6): 641, fig. 1 (2010).

TYPE: China, Hubei, Wuhan, 22 June 2001, *D*. *L*. *Fu 2001062201* (holotype: **CAF**); ibid. 15 April 2000, *T*. *B*. *Zhao & D*. *L*. *Fu 20004151* (**CAF**).

**Magnolia viridipetala** (*Y*. *W*. *Law*, *R*. *Z*. *Zhou & Q*. *F*. *Yi*) *C*. *B*. *Callaghan & SK Png* **comb. nov.**

**Basionym:** *Michelia viridipetala* Y. W. Law, R. Z. Zhou & Q. F. Yi. *In*: Q. F.Yi et al., Nordic J. Bot. 26(5–6): 338, figs 1, 2 (2008).

TYPE: China, Yunnan, Fadou Xiang, Xichou County, evergreen broad-leaved forests, 1500 m. 20 March 2001, *Ren*-*Zhang Zhou 20012* (holotype: **IBSC**).

Note – *Michelia virensipetala* Y. W. Law & R. Z. Zhou. *In*: Liu Y. H. et al. (2004): 322, is an invalid name for the same taxon, as no Latin description or diagnosis was given (ICN Art. 39.1) and no type was indicated. (ICN Art. 40.1).

**Magnolia wuzhishangensis** (*G*. *A*. *Fu & K*. *Pan*) *C*. *B*. *Callaghan & SK Png* **comb. nov.**

**Basionym:** *Michelia wuzhishangensis* G. A. Fu & K. Pan. *In*: Bull. Bot. Res., Harbin 32(3): 258, fig. 2 (2012).

TYPE: China, Hainan Island, Chiunchun County, Limushang, 1300 m. 12 Apr. 1980, *Guo*-*ai Fu 1713* (holotype: **HFB**, fl.); ibid. *Guo*-*ai Fu 0892* (**HFB**, fr.).

**Magnolia xianianhei** (*Q*. *N*. *Vu*) *C*. *B*. *Callaghan & SK Png* **comb. nov.**

**Basionym:** *Michelia xianianhei* Q. N. Vu. *In*: Nordic J. of Bot. 30(5): 575, figs. 1, 2 (2012).

TYPE: Vietnam, Dien Bien Province, Dien Bien District, Muong Phang Municipality, Vo Nguyen Giap Historical Vestige, 1004 m. 31 Dec. 2010, *Nam 311210*.*2* (holotype: **VNF**; isotype: **IBSC**).

Note – There would appear to be an irregular citation in the name of the collector of the nomenclatural type and its duplicate, since the author and collector is the same person (Vu Quang Nam). While the use of a person’s given name is customary as a primary form of address in Vietnam, its use in an international scientific journal is potentially confusing.

**Magnolia xinyangensis** (*T*. *B*. *Zhao*, *Z*. *X*. *Chen & H*. *T*. *Dai*) *C*. *B*. *Callaghan & SK Png* **comb. nov.**

**Basionym:** *Yulania xinyangensis* T. B. Zhao, Z. X. Chen & H. T. Dai. *In*: H. T. Dai, J. Li, D. L. Fu, et al., J. Xinyang Normal Uni. (Nat. Sci. Ed.) 25(4): 485, fig. 2 (2012).

TYPE: China, Henan, Xinyang Xian, Mt. Dabieshan, 25 March 2000, *T*. *B*. *Zhao et al*. *200003251* (holotype: **HNAC**); ibid. 5 Oct. 1999, *T*. *B*. *Zhao & D*. *L*. *Fu 199910051* (fol., Yulania- alabastrum).

## Conclusions

The transfer of the above eighteen taxa to *Magnolia* is necessary following the present near universal acceptance that Magnolioideae is one of two monogeneric subfamilies within Magnoliaceae and the fact that the majority of resulting new combinations and names arising from the relegation of *Manglietia* and *Michelia* into *Magnolia* have previously been made by various authors such as Figlar (2000) for the majority of *Michelia* species, Kumar (2006) for the majority of *Manglietia* species, with Sima (2001) transferring some further *Michelia* species and Nooteboom (in Xia et al. 2008:49–50) transferring a number of species from both genera.

## Authors’ original submitted files for images

Below are the links to the authors’ original submitted files for images. Authors’ original file for figure 1 Authors’ original file for figure 2
