# Supplementary figures and images for: A new name and seventeen new combinations in the Magnolia (Magnoliaceae) of China and Vietnam
Source: Bot Stud. 2013 Nov 4;54:53. doi: 10.1186/1999-3110-54-53 (PMC5432815; doi:10.1186/1999-3110-54-53)

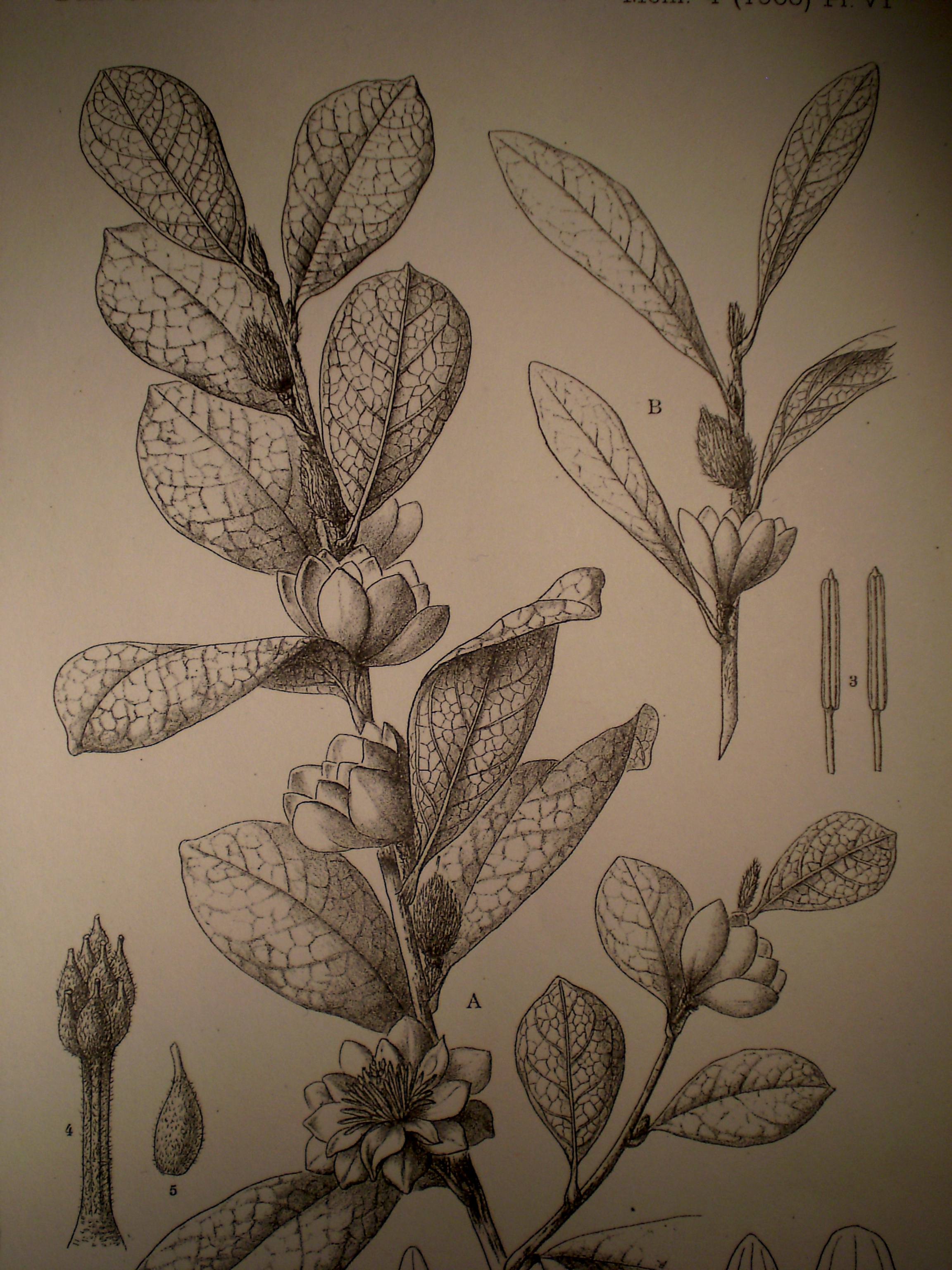

Supplement: Supplementary file 1 — Authors’ original file for figure 1 [file 40529_2013_48_MOESM1_ESM.jpeg]
